# Supplementary material for: Salivary osmolality measured by MX3 hydration testing system demonstrates high reliability but limited validity in elite athlete hydration assessment
Source: PLoS One. 2026 Jun 3;21(6):e0349955. doi: 10.1371/journal.pone.0349955 (PMC13232853; doi:10.1371/journal.pone.0349955)
Supplement: S1 File — (DOCX) [file pone.0349955.s001.docx]

**Supplementary Materials**

This file contains three supplementary tables: Table S1, Table S2, and Table S3.

S1. Categorization of included sports by sport type and number of participants (N = 118). To ensure confidentiality, counts <5 are reported as *n* < 5.

| **Sports category** | **Sport** |
| --- | --- |
| Team sport  (n = 56) | Handball (n = 34)  Ice-hockey (n = 22) |
| Endurance  (n = 22) | Athletics (n < 5)  Biathlon (n = 14)  Cycling (n < 5)  Triathlon (n < 5)  Swimming (n < 5) |
| Middle distance / Power  (n = 14) | Athletics (n < 5)  Canoe slalom (n < 5)  Canoe sprint (n = 5)  Swimming (n = 6) |
| Precision / Skill  (n = 13) | Equestrian (n < 5)  Sailing (n = 9)  Skateboard (n < 5)  Sport Climbing (n < 5) |
| Speed / Strength  (n = 7) | Athletics (n = 7) |
| Combat Sports  (n = 6) | Wrestling (n = 6) |

S2. Distribution of participants across sport categories and performance tiers.

| **Sports category** | **Tier 3 n (%)** | **Tier 4**  **n (%)** | **Tier 5**  **n (%)** | **Total**  **n** |
| --- | --- | --- | --- | --- |
| Team Sports | 0 (0%) | 30 (54%) | 26 (46%) | 56 |
| Endurance | 1 (5%) | 15 (68%) | 6 (27%) | 22 |
| Middle distance/Power | 1 (7%) | 12 (86%) | 1 (7%) | 14 |
| Precision/Skill | 0 (0%) | 9 (69%) | 4 (31%) | 13 |
| Speed/Strength | 1 (14%) | 5 (71%) | 1 (14%) | 7 |
| Combat Sports | 0 (0%) | 4 (67%) | 2 (33%) | 6 |
| Total | 3 (3%) | 75 (64%) | 40 (34%) | 118 |

S3. Cross-classification of hydration status based on USG and SOSM categories (n = 217 valid sample sets).

| USG category | Hydrated | Mildly dehydrated | Moderately dehydrated | Severely dehydrated | Total |
| --- | --- | --- | --- | --- | --- |
| Well hydrated | 1 (25%) | 2 (50%) | 1 (25%) | 0 (0%) | 4 (100%) |
| Minimal dehydration | 14 (12%) | 70 (59%) | 29 (24%) | 6 (5%) | 119 (100%) |
| Significant dehydration | 12 (13%) | 51 (57%) | 25 (28%) | 1 (1%) | 89 (100%) |
| Serious dehydration | 1 (20%) | 2 (40%) | 2 (40%) | 0 (0%) | 5 (100%) |
| Total | 28 (13%) | 125 (58%) | 57 (26%) | 7 (3%) | 217 (100%) |

*Note: USG, urine specific gravity; SOSM, salivary osmolality. Analyses include only sample sets with valid paired USG and SOSM values*
