# Supplementary material for: Salivary osmolality measured by MX3 hydration testing system demonstrates high reliability but limited validity in elite athlete hydration assessment
Source: PLoS One. 2026 Jun 3;21(6):e0349955. doi: 10.1371/journal.pone.0349955 (PMC13232853; doi:10.1371/journal.pone.0349955)
Supplement: Attachment — Submitted filename: reviwer report.pdf [file pone.0349955.s002.pdf]

Article Peer Review Report

Article Title: Salivary Osmolality Measured by MX3 Hydration Testing System Demonstrates High Reliability but Limited Validity in Elite Athlete Hydration Assessment

1. Overall Recommendation

- **Decision:** Major Revisions
- **Originality and Contribution:** Yes
- **Scientific Significance:** Yes
- **General Comment:** This study addresses a practical and relevant method for managing dehydration in elite athletes. While the study is well-structured and utilizes a high-level participant group (Olympic athletes), significant improvements are required in the methodology, statistical interpretation, and discussion of validity before publication

2. Detailed Evaluation and Revision Requirements

Abstract and Title

- **Methodology:** Briefly include the specific statistical analyses used within the abstract.
- **Findings:** The values of **ICC = 0.00** and **κ = -0.02** are critical. They suggest zero correlation between SOSM and the gold standard (USG). This needs to be highlighted clearly.
- **AUC Values:** The AUC range of **0.40–0.49** indicates predictive performance worse than chance. The abstract must reconcile this with the "excellent" test-retest reliability mentioned.
- **Conclusion:** The abstract focuses on reliability but lacks sufficient emphasis on the concerning lack of validity.

Introduction

- **Methodological Limitations:** Provide more detail on the underlying physiological causes behind the limitations of plasma osmolality and urine indices in field settings.
- **Contextualization:** Clarify if previous "controlled studies" were randomized trials or focused on acute/chronic phases. Specifically, explain why SOSM findings in pediatric/elderly populations (11, 12) do not translate to elite athletes (e.g., exercise-induced changes in saliva).
- **Timing:** Explicitly state the physiological rationale for choosing morning hydration measurements.

Methodology

- **Dietary Control:** Clarify if a dietary or fluid intake protocol was followed the previous day. If not, this must be listed as a limitation, as meal patterns impact morning hydration.

- **Mouth Breathing:** Address whether participants were screened for mouth breathing during sleep, which increases saliva viscosity and artificially inflates osmolality.
- **Sample Power:** The power calculation required **128 matched samples**, but only **118 participants** were included. Explain this shortfall or specify if any data were excluded.
- **Statistical Independence:** Since 230 samples were taken from 118 athletes (repeated measures), authors must confirm if a **mixed-effects model** was used to adjust for "within-subject" correlations.
- **Threshold Comparison:** Explain the biological overlap between the manufacturer's SOSM thresholds and the Casa et al. (15) USG references to justify the "negligible agreement" finding.

Findings

- **Nested Data:** Clarify in the statistics section how the data from participants providing multiple samples (36% provided 3 days of data) were handled to avoid violating the assumption of independent observations.
- **Accuracy:** Explicitly discuss why the AUC values (0.40–0.49) suggest the device is statistically less accurate than a random guess (0.50).
- **Categorical Discordance:** Address Table 4, where athletes labeled "significantly dehydrated" by urine analysis were classified as "hydrated" by the SOSM device.

Discussion

- **Selection Bias:** The exclusion of 13 data sets due to "high viscosity" is critical. If the most dehydrated athletes have the most viscous saliva and the device cannot read them, this creates a bias. This must be discussed.
- **Xerostomia:** Beyond circadian rhythms, consider the impact of "dry mouth" or overnight mouth breathing on saliva viscosity in the discussion.
- **Reference Standards:** Discuss whether the lack of correlation stems from SOSM failure, USG limitations (e.g., protein intake, muscle mass), or both methods failing to reflect true plasma osmolality.

Conclusion and References

- **Speculation:** The statement that SOSM "may hold value under different conditions" is speculative and not supported by the current data; it should be softened or removed.
- **Formatting:** Ensure the reference list strictly follows **PLOS ONE** guidelines.
