# Supplementary material for: Salivary osmolality measured by MX3 hydration testing system demonstrates high reliability but limited validity in elite athlete hydration assessment
Source: PLoS One. 2026 Jun 3;21(6):e0349955. doi: 10.1371/journal.pone.0349955 (PMC13232853; doi:10.1371/journal.pone.0349955)
Supplement: Attachment — Submitted filename: Article Peer Review Report.pdf [file pone.0349955.s003.pdf]

Article Peer Review Report

Article Title: Salivary Osmolality Measured by MX3 Hydration Testing System Demonstrates High Reliability but Limited Validity in Elite Athlete Hydration Assessment

Criteria	Recommendation	Notes / Comments
Overall Recommendation	[ ] Accept<br>[ ] Minor Revisions<br>[X] Major Revisions<br>[ ] Reject	
Originality and Contribution	[X] Yes [ ] Partially [ ] No	
Scientific Significance	[X] Yes [ ] Partially [ ] No	

General Suggesions

This article presents a recent study examining a more practical and feasible method for managing dehydration in elite athletes. The study is well-structured and includes results obtained from Olympic-level athletes. However, the introduction, methodology, findings, and discussion sections require improvement before the article can be published.

Detailed Evaluation Checklist

Section	Evaluation Criteria	Check (Yes/No/N/A)	Revision Needed? (Yes/No)
1. Title and Abstract	Does the title accurately and engagingly reflect the content?	[X] Yes [ ] No	[ ] Yes [X] No
	Does the abstract clearly summarize the purpose, method, findings, and conclusions?	[ ] Yes [X] No	[X] Yes [ ] No
	Are the keywords appropriate and sufficient?	[X] Yes [ ] No	[ ] Yes [X] No
	**Comments / Suggestions**		
	* The statistical analysis should be included briefly in the methods section on the abstract.<br>*The values of ICC = 0.00 and κ = -0.02 are quite striking. This suggests that there is no correlation between the SOSM value and USG, which is considered the gold standard.<br>*The fact that the AUC values range between 0.40 and 0.49 indicates moderate predictive performance; however, the results of the test-retest analysis suggest that this is actually at an excellent level, which is somewhat confusing.<br>*The conclusions section focuses on the reliability of the device, but the absence of any information regarding its validity is a cause for concern.		
2. Introduction	Is the research problem clearly defined?	[ ] Yes [X] No	[X] Yes [ ] No
	Is the literature review comprehensive and up-to-date?	[X] Yes [ ] No	[ ] Yes [ ] No
	Is the research gap sufficiently highlighted?	[X] Yes [ ] No	[ ] Yes [ ] No
	Are the study's aim/hypotheses clearly stated?	[X] Yes [ ] No	[ ] Yes [ ] No
	**Comments / Suggestions**		
	* The limitations of methods such as plasma osmolality and urine indices in practical applications have been briefly mentioned. However, more detailed information should be provided regarding the underlying causes and processes behind these limitations.<br>*'In controlled studies', were these studies randomised controlled trials, or did they examine changes in the effects of dehydration during the acute or chronic phases? In particular, if the studies concern conditions below the specified dehydration levels or dehydration induced by exercise, information regarding the use of SOSM should be provided.<br>*The authors have noted that the MX3 system has previously yielded successful results in elderly and pediatric populations (11, 12), but they could provide a more specific physiological explanation (e.g., exercise-induced changes in saliva composition) as to why these findings cannot be directly applied to healthy and physically active individuals.<br>*The study specifically chose to measure hydration levels in the morning, and it is advisable to elucidate the physiological reasons behind this decision.		

3. Method	Is the research design appropriate and clear?	[X] Yes [ ] No	[X] Yes [ ] No
	Is the sample selection and size adequate?	[X] Yes [ ] No	[X] Yes [ ] No
	Are the data collection instruments valid and reliable? Is detail provided?	[X] Yes [ ] No	[ ] Yes [ ] No
	Are the data analysis methods appropriate and correctly explained?	[X] Yes [ ] No	[X] Yes [ ] No
	Is ethical approval mentioned (if required)?	[X] Yes [ ] No [ ]	[ ] Yes [ ] No [ ]
	**Comments / Suggestions**		
	* Prior to morning measurements, it has been stated that participants were not required to follow a specific diet or fluid intake regimen the day before. However, the impact that diet and meal patterns may have on morning hydration levels in elite athletes should not be overlooked. In such cases, if a dietary questionnaire or fluid intake protocol was used, these should be included; otherwise, this limitation should be noted.<br>*Mouth breathing is common among elite athletes, particularly during sleep. This can reduce saliva flow rate and artificially increase osmolality. The method does not specify whether any observations or screening for this were carried out.<br>*A 15-minute interval has been allowed between the collection of the urine sample and the saliva sample. Whilst this may seem a reasonable period, it should be noted whether a ‘definitive’ check has been carried out to ensure that no fluids have been consumed during this time.<br>*The ‘Power Calculation’ section states that 128 matched samples are required to achieve the ICC value. However, samples were collected from only 118 participants in the study. This appears to fall short of the target. Please specify if any data or participants were excluded. If not, please explain how this shortfall in the number of matched samples was addressed.<br>*The fact that 230 samples were taken from 118 athletes implies that samples were taken from some athletes on more than one occasion (over 2 or 3 days). If an appropriate adjustment (e.g., a mixed-effects model) has been made in the analysis for these ‘within-subject’ correlations (nested data), this should be stated. Otherwise, the p-values and confidence intervals may have turned out to be narrow by chance.<br>*The measurement of the device’s internal consistency makes sense, but my advice to authors is that they should state that this ensures measurement consistency.<br>*The SOSM values were categorized using the threshold values provided by the manufacturer (MX3). However, for USG, Casa et al. (15) were used as a reference. It should be noted to what extent these two different sources overlap in order to establish the biological basis for the definitions of 'thirst.' This may have contributed to the finding of ‘negligible agreement.'		
4. Findings	Are the findings presented clearly, orderly, and understandably?	[X] Yes [ ] No	[ ] Yes [ ] No
	Are tables and figures accurate and informative?	[X] Yes [ ] No	[ ] Yes [ ] No
	Are statistical analyses correctly interpreted?	[X] Yes [ ] No	[X] Yes [ ] No
	**Comments / Suggestions**		
	In the study, 36% of participants provided data for three days, while 42% provided data for just one day. This situation may undermine the assumption of ‘independent observations’ in statistical analysis. The authors need to clarify in the statistics section whether they used a ‘mixed-effects model’ for these repeated measurements (nested data).<br>		

	The fact that the reported AUC values fall within the range of 0.40–0.49 indicates that, statistically speaking, this test is even less accurate than the threshold of 0.50. It is unclear whether this accuracy is merely random or represents a measurement specific to the device.<br>- A kappa value of 0.02 indicates low agreement between the SOSM and USG categories. However, as shown in Table 4, the fact that athletes classified as 'significantly dehydrated' based on urine analysis were categorized as 'hydrated' or 'mildly dehydrated' by the SOSM suggests that the SOSM measurement device yields different results compared to USG. This point requires clarification.		
5. Discussion	Are the findings correctly interpreted within the objectives and literature?	[ ] Yes [ ] No	[ ] Yes [ ] No
	Are the findings related to previous studies?	[ ] Yes [ ] No	[ ] Yes [ ] No
	Are the study's limitations clearly stated?	[ ] Yes [ ] No	[ ] Yes [ ] No
	Are theoretical and practical implications sufficiently highlighted?	[ ] Yes [ ] No	[ ] Yes [ ] No
	Are there suggestions for future research?	[ ] Yes [ ] No	[ ] Yes [ ] No
	**Comments / Suggestions**		
	*The exclusion of 13 data sets due to 'high viscosity' is a factor that undermines the device's reliability under real-world (field-based) conditions. If the saliva of severely dehydrated athletes is at its most viscous and the device is unable to measure it, the findings may introduce a bias in favor of dehydration. This issue needs to be addressed.<br>*They have attributed the elevated SOSM values measured immediately upon waking in the morning to the circadian rhythm. However, 'dry mouth (xerostomia)' or the effect of breathing through the mouth throughout the night on saliva viscosity could also be added to the discussion.<br>*The author has used SG (urine specific gravity) as a reference but has also discussed the limitations of USG (muscle mass, protein intake, etc.). If USG is not perfect either and does not correlate with SOSM, the confusion surrounding this situation—namely, whether both methods might be failing to accurately measure true plasma osmolality—should be clarified.<br>*They stated that they excluded 13 samples from the analysis due to their 'high viscosity.' The discussion section should address whether this situation creates a 'selection bias.'		
6. Conclusion and References	Does the conclusion section summarize key findings and takeaways?	[X] Yes [ ] No	[ ] Yes [ ] No
	Does the reference format comply with journal guidelines?	[X] Yes [ ] No	[ ] Yes [ ] No
	Are there any missing or incorrect references?	[ ] Yes [ ] No	[ ] Yes [ ] No
	**Comments / Suggestions**		
	* In the Conclusions section, the confusion in this section—where the statement that SOSM 'may hold value under different conditions' remains speculative as it is not supported by data—should be clarified.<br>* The reference list must be formatted in accordance with the PlosOne guidelines.		
	Is the language of the article fluid and clear?	[X] Yes [ ] No	[ ] Yes [ ] No

7. Language and Expression	Are there any grammar and spelling errors?	[X] Yes [ ] No	[ ] Yes [ ] No
	Is academic writing style used appropriately?	[X] Yes [ ] No	[ ] Yes [ ] No
	**Comments / Suggestions**		
	* The language of the article is clear and easy to understand.		
