# Supplementary material for: Salivary osmolality measured by MX3 hydration testing system demonstrates high reliability but limited validity in elite athlete hydration assessment
Source: PLoS One. 2026 Jun 3;21(6):e0349955. doi: 10.1371/journal.pone.0349955 (PMC13232853; doi:10.1371/journal.pone.0349955)
Supplement: Attachment — Submitted filename: Pettersson_et_al_Response_Letter_All_Reviewers_1-5).docx [file pone.0349955.s004.docx]

# **Response to Reviewers**

**Manuscript title:** Salivary Osmolality Measured by MX3 Hydration Testing System Demonstrates High Reliability but Limited Validity in Elite Athlete Hydration Assessment

**Journal:** PLOS ONE

We thank all five reviewers for their careful and constructive evaluation of our manuscript. Below we provide point-by-point responses to each reviewer. All changes to the manuscript are described in context. Revised passages are indicated in the tracked-changes version submitted alongside this letter.

**Reviewer 1 (Nathan Bartman)**

Thank you for your positive assessment and constructive feedback. We have addressed all of your comments and provide point-by-point responses below.

**Point-by-point responses**

*Comment 1. An important consideration is that this study assessed two different measurement tools for two different body fluids, simultaneously testing device accuracy and the usefulness of saliva to reflect urinary hydration status. Based on these results, it might be more appropriate to label saliva as inconsistent for showing hydration status. In other words, if the device were used to test urine, would it have closer agreement with USG?*

**Response.** We agree that this is an important conceptual distinction and have incorporated it more explicitly into the Discussion. The study simultaneously tests two things: (1) whether the MX3 device reads SOSM consistently (which it does, as shown by the high within-sample ICC), and (2) whether SOSM as a biomarker reflects the same information as USG (which it does not, as shown by the near-zero validity ICC and negligible kappa). We now state more clearly that the poor agreement likely reflects the unsuitability of first-morning saliva as a hydration matrix in this context, rather than device malfunction per se. Regarding the hypothetical of using the device to measure urine: this falls outside the device's intended measurement principle (electrochemical impedance spectroscopy of saliva) and we therefore do not speculate further on this in the manuscript.

*Comment 2. Abstract (L30–31): A kappa value <0 might be better described as "no agreement" rather than "negligible".*

**Response.** We agree. A negative kappa value falls below the lowest interpretable category in the McHugh scale. We have revised the abstract to read "no meaningful classification agreement (κ = −0.02; 95% CI [−0.15, 0.10])", which more accurately reflects both the magnitude and the statistical uncertainty of this finding. Because the confidence interval spans zero, we cannot conclude that the value is meaningfully different from chance-level agreement.

*Comment 3. Abstract (L74): Did you confirm that participants were fasted, or did you control the amount of time fasted?*

**Response.** Participants were instructed to remain fasted from bedtime until saliva collection was completed, but fasting duration was not formally quantified and adherence was self-reported rather than independently verified. We have added this clarification to the Methods section, and it is also acknowledged as a study limitation.

*Comment 4. Introduction (L150, L160): Period appears before the reference.*

**Response.** Corrected in the revised manuscript.

*Comment 5. Methods — Hydration Classification: It is unclear whether participants need to fit both SOSM and USG criteria for classification. The USG categories should be defined explicitly.*

**Response.** We have revised the Hydration Classification section to clarify that SOSM and USG categories are assigned independently using separate source-specific thresholds. A participant is classified according to each method separately, not by a joint criterion. The USG categories (well hydrated ≤1.010, minimal dehydration 1.011–1.020, significant dehydration 1.021–1.030, and serious dehydration ≥1.031) are now defined explicitly in the Methods.

*Comment 6. Results — Table 2: Some sub-group proportions add up to 100.8%. It is also unclear why USG has both ordinal categories and cumulative binary thresholds, and whether these are mutually exclusive.*

**Response.** We have corrected the rounding in Table 2. The ordinal categories and the binary threshold categories serve different analytical purposes. We have added a table note clarifying that the binary threshold values represent cumulative proportions and are not mutually exclusive.

*Comment 7. Discussion: The SOSM threshold for USG ≥1.025 is 76 mOsm, which is lower than the 84 mOsm for USG ≥1.020. Can you clarify why?*

**Response.** The SOSM thresholds were derived empirically from ROC curves by maximizing Youden's index and are therefore data-driven rather than physiologically ordered. The non-monotonic pattern (76 mOsm for USG ≥1.025 vs. 84 mOsm for USG ≥1.020) likely reflects the low prevalence of samples exceeding USG ≥1.025 (13%), which reduces the stability of the Youden-optimized estimate. Given that this pattern does not affect the overall interpretation, as AUC values near 0.50 and kappa values near zero across all thresholds indicate uniformly poor discrimination, we did not add explanatory text to the manuscript.

*Comment 8. Discussion (L258): To enable equal comparison with Atjo et al. (r = 0.78), what was the Pearson r value in the present study?*

**Response.** The Pearson correlation between SOSM and USG in our sample was r = −0.01 (95% CI [−0.14, 0.12]), consistent with ICC = 0.00. We have added this value to the existing validity statement in the Discussion to enable direct comparison with Atjo et al.

*Comment 9. Discussion (L265): Rephrase "USG cut-offs" as "USG categories" to avoid implying a single threshold.*

**Response.** Corrected. We have revised the relevant passage in the Discussion to read "USG threshold" rather than "USG cut-off" to avoid implying that classification is based on a single value rather than a defined range.

*Comment 10. Since MDC80 and MDC90 values were reported, can you explain what these mean to the average user?*

**Response.** We have added a brief clarification to the Results, noting that repeated readings must differ by at least the MDC value to exceed measurement error. Specifically, the text now reads that the MDC90 of 18.4 mOsm indicates that a difference between two repeated readings must exceed this value to be considered a true change rather than measurement error, with 90% confidence.

*Comment 11. Discussion (L305): Change "was" to "were".*

**Response.** Corrected.

*Comment 12. Limitations: Why was serum (plasma osmolality) not analyzed?*

**Response.** Plasma osmolality was not feasible given that data collection took place at remote international training camps where venous blood sampling and laboratory analysis were logistically not possible. The manuscript notes that plasma osmolality was not feasible in the camp-based setting, and the limitations section now explicitly acknowledges that neither plasma osmolality nor body mass change was assessed, precluding definitive conclusions about whether the discordance reflects failure of SOSM, limitations of USG, or both.

Sincerely,

The authors

**Reviewer 2 (Jacobus P. Van Wouwe)**

Thank you for your positive appraisal and constructive comments. We address each point below.

**Point-by-point responses**

*Comment 1. It is unclear whether there were any missing values or participants willing to join who actually did drop out.*

**Response.** All 118 athletes who provided written informed consent completed the study protocol; there were no dropouts. Across the 230 sampling occasions, 13 saliva sample sets were excluded because the MX3 device did not return a readable value, and these are now reported explicitly in the Results. No participants withdrew and no urine samples were missing.

*Comment 2. The differences in weather conditions across collection sites (Fuerteventura in February, Font-Romeu in October, Stockholm in November) have not been taken into account. Could environmental conditions influence SOSM differently than USG? Are results for samples from the three locations identical?*

**Response.** Since USG and SOSM were collected simultaneously under the same morning conditions at each site, differential environmental effects on the two measures are unlikely to explain the observed discordance — both biomarkers were exposed to the same ambient conditions. Site-specific subgroup analyses were not performed as sport type was confounded with location and the study was not powered for such comparisons. We therefore did not add this as a formal limitation in the manuscript.

*Comment 3. In the Results, you report no sex difference in USG. This seems also to be the case for SOSM — why not add it?*

**Response.** We agree and have added the sex comparison for SOSM to the Results. The revised manuscript now states that no significant sex difference was observed in either USG or SOSM (p > 0.05 for both).

Sincerely,

The authors

**Reviewer 3**

Thank you for the thorough and thoughtful review. Your twelve questions have helped us sharpen several aspects of the manuscript. We respond to each question below.

**Point-by-point responses**

*Question 1. Why was urine specific gravity selected as the sole reference method, given that it is not considered the gold standard?*

**Response.** We acknowledge that USG is not the gold standard. However, plasma osmolality was not feasible given the absence of laboratory infrastructure at the remote training venues. USG was selected because it is the most widely used field-based hydration reference in applied sport settings, recommended by major sports medicine organizations for first-morning monitoring, and supported by prior validation against plasma osmolality. We have added an explicit justification for this choice in the Discussion, along with an acknowledgment of USG's own limitations in athletic populations.

*Question 2. Did the authors consider including plasma osmolality or body mass changes as additional validation markers?*

**Response.** Yes, we considered both. Plasma osmolality was not logistically feasible, as described above. Body mass change was not measured because the study focused on first-morning classification of hydration status rather than acute fluid balance tracking, and because reference body mass in the euhydrated state was not available for these athletes at the time of measurement. Both omissions are acknowledged in the limitations section of the revised manuscript.

*Question 3. How was the potential influence of circadian rhythm on salivary osmolality controlled?*

**Response.** Circadian variation was standardized by design: all samples were collected within a fixed morning window (06:00–08:00) under fasted conditions, minimizing within-individual temporal variation. However, we cannot exclude that circadian effects — such as reduced salivary flow and elevated osmolality upon waking — inflated SOSM values systematically in the morning. We discuss this explicitly in the Discussion as a potential explanation for the high prevalence of SOSM readings classified as dehydrated relative to USG.

*Question 4. Could the lack of intentional hydration manipulation have limited the range of observed values and thereby affected diagnostic validity?*

**Response.** Yes, this is discussed in the Discussion. Free-living conditions maximized ecological validity but resulted in a cohort that was generally well-hydrated: only 13% exceeded USG ≥1.025. A restricted range of hydration states reduces the statistical signal available for discrimination, which likely contributed to the poor diagnostic accuracy of SOSM in this sample. This is contrasted with the Atjo et al. cohort, where 89% exceeded USG 1.025 and where stronger agreement was reported.

*Question 5. How do you interpret the finding that most samples were classified as dehydrated by SOSM while USG indicated normal or mildly reduced hydration?*

**Response.** We interpret this as reflecting circadian and local oral factors rather than true whole-body dehydration. Salivary flow is at its diurnal nadir in the morning and solute concentration is elevated, which may inflate SOSM independently of systemic hydration. Overnight oral drying from mouth breathing, snoring, or xerostomia may further concentrate saliva locally. These factors are discussed in the physiological explanation section of the Discussion.

*Question 6. Is it possible that discrepancies arise from physiological differences in saliva and urine regulation rather than purely methodological limitations?*

**Response.** Yes, and we now state this explicitly. USG reflects renal concentration of solutes over the preceding hours, whereas SOSM reflects the composition of oral fluid at the moment of sampling, regulated by salivary gland secretion, circadian rhythms, sympathetic tone, and local oral conditions. These are physiologically distinct compartments, and we clarify in the Hydration Classification section and Discussion that agreement was assessed as an empirical question rather than assumed on the basis of biological equivalence.

*Question 7. Did the authors conduct subgroup analyses by sex, sport discipline, or performance level?*

**Response.** Formal subgroup analyses by sport discipline and performance tier were not conducted. There is no established physiological mechanism by which SOSM validity would be expected to differ systematically across sport types or competitive levels, and the number of athletes in several sport-specific subgroups was too small for meaningful analysis. Sex differences in both USG and SOSM were examined and are now reported in the Results; no significant differences were found for either measure.

*Question 8. To what extent might saliva viscosity and unsuccessful device readings have influenced the results, and was a sensitivity analysis performed?*

**Response.** The 13 excluded sample sets represent cases where the MX3 device returned no readable value, most plausibly due to elevated saliva viscosity. We discuss the potential for selection bias explicitly: if highly viscous or concentrated saliva was disproportionately unreadable, the exclusions may have removed precisely those cases where the device would perform most poorly. A formal sensitivity analysis was not possible because the excluded cases lack SOSM values. The potential direction of this bias is acknowledged in the Discussion and limitations.

*Question 9. Are the manufacturer's SOSM classification thresholds appropriate for elite athletes, or should they be calibrated specifically for this population?*

**Response.** It is physiologically unlikely that fasted morning saliva composition in elite athletes would differ substantially from that of other healthy young adults, suggesting that the manufacturer thresholds themselves may not be the primary explanation for the poor classification agreement. Rather, our findings suggest that first-morning saliva is an unsuitable matrix for hydration classification in this context, regardless of the thresholds applied. Population-specific validation under different sampling conditions would be needed before any practical application in athletes could be justified.

*Question 10. How do the authors envision potential applications of SOSM under alternative conditions, such as post-exercise or during training?*

**Response.** We deliberately avoid speculating about alternative conditions given the absence of supporting data. The Conclusion states that future studies should examine SOSM under other sampling conditions before any practical role can be inferred.

*Question 11. Could repeated measurements from the same saliva sample have artificially inflated the reliability estimates compared to real-world use?*

**Response.** Yes, and we now make this distinction explicit in both the Results and Discussion. Repeated readings of the same sample eliminate variation from collection technique, volume, and transient oral factors. Our ICC of 0.93 therefore represents the upper bound of device repeatability under optimal conditions, as we acknowledge by contrasting it with the lower ICC (0.75) reported by Winter et al., who collected two separate saliva samples.

*Question 12. What are the practical implications for coaches and medical staff who already use the device?*

**Response.** The principal practical implication is that first-morning SOSM measured with the MX3 HTS should not be used as a standalone hydration indicator in elite athletes under free-living conditions. The device can read SOSM consistently, but the values do not correspond to USG-based hydration categories in this population and context. We have reinforced this implication in the Conclusion.

Sincerely,

The authors

**Reviewer 4 (reviwer report.pdf)**

Thank you for your careful reading and the detailed and structured evaluation. We have revised the manuscript substantially in response to your suggestions. Below we provide a point-by-point response describing how each comment has been addressed.

**Point-by-point responses**

*Comment 1. Please briefly include the specific statistical analyses used within the abstract, and make the lack of validity more explicit.*

**Response.** The abstract has been revised to state the main analyses used for validity and repeatability, specifically ICC(3,1), ROC analysis, and Cohen's kappa. We also made the lack of validity more explicit by stating that SOSM showed no meaningful correspondence with USG, that classification agreement was negligible, and that diagnostic accuracy was poor across all examined threshold comparisons, whereas the high repeatability result applied only to repeated readings from the same saliva sample.

*Comment 2. The values of ICC = 0.00, κ = −0.02, and AUC = 0.40–0.49 indicate very poor validity and should be reconciled with the reported excellent reliability.*

**Response.** We now distinguish validity from within-sample repeatability more clearly throughout the manuscript. In the abstract, Results, Discussion, and Conclusion, we explicitly state that the AUC values indicate chance-level to worse-than-chance discrimination, while the reliability result refers only to repeated measurements of the same saliva sample under standardized conditions. The abstract has also been revised to describe the kappa as "no meaningful classification agreement (κ = −0.02; 95% CI [−0.15, 0.10])", as the confidence interval spans zero and we cannot conclude the value is meaningfully different from chance.

*Comment 3. Provide more detail on the physiological limitations of plasma osmolality and urine indices in field settings.*

**Response.** The Introduction has been expanded accordingly. We now state that plasma osmolality is tightly homeostatically regulated, typically changes only with more pronounced fluid deficits, and is impractical for routine athlete monitoring because it requires venous blood sampling and laboratory analysis. We also clarify that urine indices reflect the renal response to recent fluid and solute intake over preceding hours rather than an immediate snapshot of total-body water.

*Comment 4. Clarify the context of previous controlled SOSM studies and explain why findings in pediatric/elderly cohorts may not translate to elite athletes.*

**Response.** We revised the Introduction to specify that the supportive controlled SOSM studies involved acute exercise-induced dehydration or short-term fluid restriction rather than routine free-living monitoring. We also clarified why direct translation from pediatric and elderly clinical cohorts to elite athletes is limited, emphasizing differences in age, health status, medication use, and athlete-specific oral exposures such as oral breathing, carbohydrate intake, and repeated mouth rinsing.

*Comment 5. Explicitly state the physiological rationale for choosing morning hydration measurements.*

**Response.** We added this rationale to the Introduction. The revised text now explains that standardized conditions such as overnight fasting minimize external influences and that first-morning timing is particularly relevant in athlete monitoring because it reflects hydration status at the start of the day, before substantial behavioral and environmental variation accumulates.

*Comment 6. Clarify whether a dietary or fluid-intake protocol was followed the previous day, and if not, list this as a limitation.*

**Response.** The Methods section now states explicitly that no standardized diet or fluid-intake protocol was imposed on the preceding day and that participants were asked to follow their normal routines. This point is also acknowledged in the limitations section.

*Comment 7. Address whether participants were screened for mouth breathing during sleep.*

**Response.** We now state in the Methods that no formal information was collected on nocturnal mouth breathing, snoring, nasal congestion, xerostomia, or other potential causes of overnight oral drying. The Discussion was expanded accordingly to note that these factors could further concentrate oral fluid independently of whole-body hydration and were not assessed in the present study.

*Comment 8. The power calculation required 128 matched samples, but only 118 participants were included. Please explain this.*

**Response.** We clarified that the power target referred to paired measurement occasions rather than unique athletes. The Methods now state this explicitly and note that the final analytic sample of 230 paired sample sets exceeded the a priori target of 128.

*Comment 9. Because 230 samples were obtained from 118 athletes, please clarify how repeated measures were handled and whether a mixed-effects model was used.*

**Response.** The Statistics section now states explicitly that analyses were conducted at the sample-set level and that no mixed-effects model or other adjustment for within-subject clustering was applied. Interpretation therefore focused on the magnitude and direction of the agreement and accuracy metrics rather than on statistical significance alone. This is also acknowledged as a limitation.

*Comment 10. Explain the biological overlap between the manufacturer's SOSM thresholds and the Casa et al. USG references.*

**Response.** The Hydration Classification subsection has been revised to clarify that the study did not treat SOSM and USG as physiologically equivalent measures. We now explain that USG and SOSM reflect different physiological processes, and that the comparison was intended to assess empirical alignment between field-based classification systems rather than biological equivalence. We also explain that thresholds of 1.020, 1.025, and 1.030 were examined given the lack of consensus regarding the most appropriate USG cut-off, and that corresponding SOSM thresholds were determined empirically from the data.

*Comment 11. Clarify how the nested data were handled in the Results and discuss why AUC values below 0.50 indicate performance worse than random guessing.*

**Response.** We clarified in the Statistics section that repeated observations were analyzed at the sample-set level. In the Results, the threshold section has been rewritten to state explicitly that AUC values of 0.40–0.49 indicate discrimination no better than or worse than chance.

*Comment 12. Address Table 4, where athletes labeled significantly dehydrated by urine were classified as hydrated by SOSM.*

**Response.** We now address this discordance explicitly in the Results text, stating that among the 89 samples classified as significantly dehydrated by USG, 12 (13%) were classified as hydrated by SOSM. The complete cross-classification data have been moved to Supplementary Table S3 to streamline the main manuscript.

*Comment 13. Discuss the potential selection bias introduced by excluding 13 unreadable/high-viscosity saliva samples.*

**Response.** We expanded both the Results and the Discussion on this point. We now state explicitly that 13 sampling events were excluded because the MX3 device did not return a readable SOSM value, and we discuss that if highly viscous or concentrated saliva was disproportionately unreadable, this could introduce selection bias by removing precisely those cases in which the device may be most challenged under field conditions.

*Comment 14. Consider the impact of xerostomia or overnight mouth breathing in addition to circadian rhythms.*

**Response.** This point has been incorporated into the Discussion. In addition to circadian changes in salivary composition, we now state that overnight xerostomia, mouth breathing, snoring, or nasal obstruction could further concentrate oral fluid independently of whole-body hydration and may therefore have contributed to the poor agreement with USG.

*Comment 15. Discuss whether the lack of correlation stems from SOSM failure, USG limitations, or both methods failing to reflect true plasma osmolality.*

**Response.** We expanded the Discussion to address this issue directly. The revised manuscript acknowledges that USG is not without limitations in athletic populations and can be influenced by recent protein intake, muscle mass, training stress, and sampling time. Because plasma osmolality was not measured, we now state explicitly that the present design cannot determine whether the observed discordance reflects failure of SOSM, limitations of USG, or some degree of error in both methods.

*Comment 16. The statement that SOSM "may hold value under different conditions" is speculative. Also ensure the reference list follows PLOS ONE guidelines.*

**Response.** We revised the concluding section to remove this speculative wording. The manuscript now states that future studies should examine SOSM under other sampling conditions before any practical role can be inferred. The reference list was also checked and standardized to align with PLOS ONE formatting requirements.

Sincerely,

The authors

**Reviewer 5 (Article Peer Reviwer Report.pdf)**

Thank you for your careful and constructive review. We have revised the manuscript in response to all comments and provide point-by-point responses below.

**Point-by-point responses**

*Comment 1. The abstract should briefly identify the statistical analyses used, clarify the strikingly poor validity findings, and better reconcile these with the reported excellent test-retest reliability.*

**Response.** The abstract has been revised to identify the principal analyses used for validity and reliability, specifically ICC(3,1), ROC/AUC analysis, and Cohen's kappa. We also made the lack of validity more explicit. In addition, the abstract now describes the kappa as "no meaningful classification agreement (κ = −0.02; 95% CI [−0.15, 0.10])" rather than "negligible", as the confidence interval spans zero and a negative kappa falls below the lowest interpretable category in the McHugh scale. The abstract now also distinguishes clearly between poor validity against the reference method and high within-sample repeatability when the same saliva sample is read repeatedly under standardized conditions.

*Comment 2. The introduction should explain the practical limitations of plasma osmolality and urine indices in greater physiological detail.*

**Response.** We expanded the opening paragraph of the Introduction to explain that plasma osmolality is tightly homeostatically regulated, tends to change mainly when fluid deficits become more pronounced, and requires venous blood sampling with laboratory analysis. We also clarified that urine indices primarily reflect the renal response to recent fluid and solute intake over preceding hours rather than an immediate snapshot of total-body water.

*Comment 3. Please clarify the context of the previous controlled SOSM studies and explain why findings from pediatric and elderly clinical populations may not translate to elite athletes.*

**Response.** The Introduction now specifies that the supportive SOSM studies examined either acute exercise-induced dehydration or short-term fluid restriction under controlled physiological conditions. We also revised the text to explain that direct generalization from pediatric and elderly clinical cohorts to elite athletes is limited by differences in age, health status, medication use, and athlete-specific training and competition exposures that may affect saliva composition.

*Comment 4. The physiological rationale for choosing morning measurements should be made clearer.*

**Response.** We revised the Introduction to explain that first-morning sampling reflects hydration status at the start of the day under relatively standardized conditions, before substantial daytime behavioral and environmental variation accumulates. The rationale is now presented as part of the scientific background rather than as a post hoc defense of the chosen method.

*Comment 5. Please clarify whether any dietary or fluid-intake protocol was used on the preceding day, and if not, acknowledge this as a limitation.*

**Response.** The Methods now state explicitly that no standardized diet or fluid-intake protocol was imposed on the preceding day and that participants followed their normal routines so that sampling would reflect free-living morning conditions. This is also identified as a limitation in the Discussion.

*Comment 6. The manuscript should address mouth breathing, snoring, xerostomia, or other causes of overnight oral drying.*

**Response.** We now state in the Methods that no formal information was collected on nocturnal mouth breathing, snoring, nasal congestion, xerostomia, or other potential causes of overnight oral drying. In the Discussion, we note that these factors may have increased morning saliva concentration independently of whole-body hydration and therefore may have contributed to the observed discordance between SOSM and USG.

*Comment 7. The 15-minute interval between urine and saliva sampling should be clarified, including whether participants could have consumed food or fluid during this time.*

**Response.** The revised Methods state that participants were instructed to remain fasted until saliva collection was completed, making clear that no eating or drinking was permitted during this interval. Adherence was self-reported and not independently verified, which is acknowledged as a limitation.

*Comment 8. The power calculation required 128 matched samples, whereas only 118 athletes were included. Please explain this.*

**Response.** The Power Calculation section now clarifies that the target referred to paired measurement occasions rather than unique athletes. Although 118 athletes participated, repeated sampling over one to three mornings yielded 230 paired sample sets, exceeding the required number of paired observations. We also report explicitly that 13 sampling events were excluded because the MX3 device did not return a readable SOSM value.

*Comment 9. Because 230 sample sets were obtained from 118 athletes, please clarify how repeated observations were handled and whether a mixed-effects model was used.*

**Response.** The Statistics section now states explicitly that analyses were performed at the sample-set level and that no mixed-effects model or other adjustment for within-subject clustering was applied. We added a corresponding limitation in the Discussion noting that this may have led to somewhat optimistic precision estimates, although it is unlikely to alter the overall interpretation given the consistently near-zero agreement and poor discrimination.

*Comment 10. The manuscript should state clearly that the repeatability analysis reflects measurement consistency of the device.*

**Response.** We made this distinction more explicit throughout. In the reliability results and subsequent interpretation, we now clarify that repeated within-sample SOSM readings reflect device repeatability — measurement consistency when the same saliva sample is read repeatedly under standardized conditions — rather than validity against the urine-based reference.

*Comment 11. The relationship between the manufacturer-defined SOSM categories and the Casa et al. USG thresholds should be clarified, especially regarding biological overlap.*

**Response.** The Hydration Classification section has been revised substantially. The text now states that the study did not assume biological equivalence between SOSM and USG. Although both systems rank hydration status ordinally, they capture different physiological processes, and the comparison was intended to assess empirical alignment between two field-based classification systems rather than biological equivalence between the underlying markers.

*Comment 12. The Results and Discussion should clarify how the repeated data structure affects interpretation, and the manuscript should explain the meaning of AUC values below 0.50.*

**Response.** We now report the repeated sampling structure clearly in the Results. We also revised the threshold-performance text so that AUC values of 0.40–0.49 are interpreted explicitly as chance-level to worse-than-chance discrimination.

*Comment 13. The categorical discordance between USG and SOSM requires clarification, particularly where athletes classified as significantly dehydrated by USG were classified as hydrated or mildly dehydrated by SOSM.*

**Response.** We added an explicit cross-classification result showing that among the 89 samples classified as significantly dehydrated by USG, 12 (13%) were classified as hydrated by SOSM and most were categorized as only mildly dehydrated. The Discussion explains the practical implication — that SOSM could provide false reassurance by classifying some athletes with elevated first-morning USG as hydrated.

*Comment 14. The exclusion of 13 samples due to high viscosity should be addressed as a potential selection bias.*

**Response.** This issue is now addressed explicitly in both the Results and Discussion. We report the 13 exclusions and discuss that if highly viscous or concentrated saliva was disproportionately unreadable, this may have introduced selection bias by removing precisely those cases in which the device would be most challenged under real-world conditions.

*Comment 15. If USG is not perfect either and does not correlate with SOSM, clarify whether both methods might be failing to measure true plasma osmolality.*

**Response.** We expanded the Discussion to address this directly. The revised manuscript acknowledges that USG can be influenced by recent protein intake, muscle mass, and training stress. Because plasma osmolality was not measured, we now state explicitly that the present design cannot determine whether the discordance reflects failure of SOSM, limitations of USG, or some degree of error in both methods.

*Comment 16. The statement that SOSM "may hold value under different conditions" is speculative. The reference list must follow PLOS ONE guidelines.*

**Response.** We revised the concluding section to remove this speculative wording. The manuscript now states that future studies should examine SOSM under other sampling conditions before any practical role can be inferred. The reference list was also checked and standardized to align with PLOS ONE formatting requirements.

Sincerely,

The authors
